# HIV recency testing: should results be disclosed to individuals tested?

**DOI:** 10.1002/jia2.25584

**Published:** 2020-08-26

**Authors:** Quarraisha Abdool Karim, Ruth Macklin, Sofia Gruskin, Sara Klucking, Lejeune Lockett, Celia J Maxwell, Kenneth H Mayer, Edwin Sanders, Frederick Sawe

**Affiliations:** ^1^ Centre for the AIDS Programme of Research in South Africa (CAPRISA) Durban South Africa; ^2^ Department of Epidemiology Columbia University New York NY USA; ^3^ Department of Epidemiology & Population Health Albert Einstein College of Medicine New York NY USA; ^4^ USC Institute on Inequalities in Global Health University of Southern California Los Angeles CA USA; ^5^ Office of the U.S. Global AIDS Coordinator and Health Diplomacy Washington DC USA; ^6^ Office of International Affairs Charles R Drew University of Medicine and Science Los Angeles CA USA; ^7^ Infectious Disease Clinic Howard University Hospital Washington DC USA; ^8^ Department of Medicine Beth Israel Deaconess Medical Center/Harvard Medical School and Fenway Community Health Center Harvard T.H. Chan School of Public Health Boston MA USA; ^9^ Metropolitan Interdenominational Church of Nashville Nashville TN USA; ^10^ Kenya Medical Research Institute Kericho Kenya

**Keywords:** HIV, surveillance, recency, prevention, assay

Preventing new HIV infection remains a major challenge in our response to the HIV epidemic. A diagnostic assay that could rapidly identify acutely infected individuals who are not yet antibody positive could transform our response to the epidemic, particularly in high burden countries and communities. At an individual level, a positive recency test result would need to be confirmed with a more definitive assay, and that could lead to the individual being immediately offered to initiate antiretroviral therapy. Diagnosing individuals who have recently acquired HIV, initiating them on treatment and achieving viral transmission, reduces the likelihood of onward transmission at a time when viral load is highest, but routinely used antibody assays are unlikely to find them. The identification of new or unknown HIV infections through recency testing together with geo‐spatial mapping, could enable prioritization of HIV prevention and treatment efforts to communities with increasing new transmissions. Information on those who have been recently infected or with unknown HIV status could be especially helpful for achieving the first two ‘90’s of the UNAIDS 2020 ‘90‐90‐90’ strategy: prioritization of HIV testing and linkage to care in addition to the public health benefits of reducing HIV transmission.

The recency assay currently being used in population‐based surveys in sub‐Saharan Africa and as part of laboratory surveillance and in clinical care in some settings in the USA, namely the lag avidity assay [1], uses an antibody‐based algorithm that primarily differentiates between an infection acquired less than a year ago versus more than a year ago. The data generated from the use of this assay provide some insights on annual temporal trends in new HIV infections for intervention and resource prioritization at a geo‐spatial level; however, it provides very little additional benefit to the individual beyond what available and accessible testing and treatment options already in place offer [2].

With increasing use of the current recency assay and as new assays are developed, disclosure of the results to the individual needs to be carefully considered, taking risks and benefits into account. Given the ongoing high rates of HIV stigma and discrimination, empiric evidence regarding experiences of disclosure of recency results at an individual and/or community level needs to be garnered in consultation with affected communities and key stakeholders prior to decisions to disclose results.

Another use of recency testing particularly in concentrated and generalized epidemics is for the index case to participate in voluntary assisted partner notification and identify other individuals in their network. In this situation, implementation staff need to be mindful that learning that one has acquired HIV is an emotional experience, even without the added burden of having to disclose the names of individuals in one’s sexual network. Importantly, the voluntary nature of disclosure of partner(s) must be underscored to the individual. In a clinical care context, this will require adequate training and monitoring of clinical care staff undertaking recency testing. Moreover, assisted partner notification services can be provided to newly diagnosed individuals without requiring that they know how recently they were infected.

Additional considerations relating to disclosure of recency test results arise from the social, legal and ethical factors that vary among countries: criminalization of HIV transmission or non‐disclosure of HIV status; specific behaviours and sexual identity that may be stigmatized, criminalized, or otherwise illegal and gender power disparities. Given the diversity in epidemic typology; the magnitude of the epidemic; the numerous populations at risk; legal, social and political environments and the level of preparedness of users and providers, the benefits of disclosure to individuals of test results are likely to vary. Consultations with all relevant stakeholders, including members of vulnerable populations, are essential to informing policy decisions on disclosure of recency testing results to individuals. This is not new in HIV [3, 4, 5, 6, 7], but while some of the disparities and inequities remain, knowledge of HIV status can enable effective steps to prevent transmission to sexual or needle‐sharing partners to be taken. There are still some government entities, however, that use the individual and partner or network data to stigmatize, discriminate, or engage in other violations of rights, whether or not the results are provided directly to the individuals [8, 9].

Given the uncertainties and difficulties in determining whether the potential benefits outweigh the risks, it would be premature to issue a blanket recommendation on the appropriateness of returning recency test results to individuals with the lag avidity assay. However, as recency assays evolve, the future may bring an assay able to identify acutely infected individuals who are not yet antibody positive. At an individual level, the positive test result would need to be confirmed with a more definitive assay, and if confirmed, the individual could be immediately offered to initiate antiretroviral therapy. The individual and public health benefits that could accrue from disclosure to individuals of recency test results, along with better information and approaches to safeguarding rights and addressing other gaps in current knowledge, may lead to reconsidering decisions on disclosure of recency test results to the individual or policy makers and programme managers.

## COMPETING INTERESTS

The authors declare no conflict of interest.

## AUTHORS’ CONTRIBUTIONS

All authors are members of the PEPFAR Scientific Advisory Board Recency Testing Expert Working Group and contributed to the content of this viewpoint. QAK and RM contributed equally to preparing the first draft of this viewpoint. SG, SK, LL, CM, KM, ES and FS reviewed and provided inputs on the draft. All authors have read and approved the final manuscript.
